# Study protocol of the SWORD-study: a randomised controlled trial comparing combined online and face-to-face cognitive behaviour therapy versus treatment as usual in managing fear of cancer recurrence

**DOI:** 10.1186/s40359-015-0068-1

**Published:** 2015-04-16

**Authors:** Marieke A van de Wal, Marieke FM Gielissen, Petra Servaes, Hans Knoop, Anne EM Speckens, Judith B Prins

**Affiliations:** Department of Medical Psychology, Radboud University Medical Center, (840), P.O. Box 9101, , NL - 6500 HB Nijmegen, The Netherlands; Radboud University Medical Center, Expert Centre for Chronic Fatigue, Nijmegen, The Netherlands; Department of Psychiatry, Radboud University Medical Center, Nijmegen, The Netherlands

**Keywords:** Fear of cancer recurrence, Cancer survivors, Intervention, Cognitive behaviour therapy, Randomised controlled trial, Oncology

## Abstract

**Background:**

Fear of cancer recurrence (FCR) is one of the most frequently cited problems by cancer survivors. More than one third report high FCR, which is a clinical concern due to its association with negative health outcomes. The aim of the current study is to evaluate the efficacy of cognitive behaviour therapy (CBT) in reducing FCR in high fearful cancer survivors.

**Methods/design:**

The SWORD-study has a randomised controlled design with two arms. A sample of 104 high fearful cancer survivors (breast, prostate or colorectal cancer) will be recruited from local hospitals. Cancer survivors will be randomised to receive CBT (intervention condition) or treatment as usual (control condition). For those in the intervention condition, the therapy will be individually delivered in a combination of 5 face-to-face therapy sessions and 3 online or telephone sessions by a trained therapist. Furthermore, these survivors will have access to a supportive website (or workbook) throughout the therapy. Survivors in the control condition will not receive the intervention and will not have access to the website. The primary outcome will be severity of fear of recurrence (Cancer Worry Scale). Quality of life (EORTC Quality of Life Questionnaire Core 30) and general psychological wellbeing will be assessed as secondary outcomes. Assessments will take place at baseline (before random assignment), at 3, 9 and 15 months after the baseline assessment. The study has been approved by an ethical review board.

**Discussion:**

If the intervention proves to be effective an evidence-based therapy to manage high FCR will become available for use in clinical practice.

**Electronic supplementary material:**

The online version of this article (doi:10.1186/s40359-015-0068-1) contains supplementary material, which is available to authorized users.

## Background

The number of people diagnosed with cancer is steadily increasing while medical advancements have significantly decreased cancer mortality rates. In the period beyond diagnosis and active treatment cancer survivors are faced with several emotional challenges. Handling fear of cancer recurrence (FCR) is one of the most prominent ones (Crist & Grunfeld 2012).

FCR is defined as ‘the fear or worry that the disease will return or progress in the same organ or in another part of the body’ (Vickberg 2003). FCR is a universal concern that manifests itself on a continuum, with mild uncertainty and worry on one end to severe FCR on the other end. A certain level of FCR is therefore considered normal and may even be functional; it motivates appropriate self-protective responses (e.g. staying alert for signs of a potential recurrence). However, high FCR can detrimentally affect a survivors’ emotional wellbeing (Simard et al. 2013) and may persist for years after completion of medical treatment (Savard & Ivers 2013; Deimling et al. 2006).

Moderate to high FCR is present in about 30 to 70% of cancer survivors (Savard & Ivers 2013; Custers et al. 2014; Thewes et al. 2012; Simard & Savard 2007). High FCR represents a form of distress related to the illness and aspects of treatment e.g. the cancer itself, follow-up care or periodic examinations. It is also related to psychosocial concerns such as worries about the future, disability or death (Mehnert et al. 2009). FCR is in the top five of greatest concerns for cancer survivors and has consistently been identified as one of the most cited unmet needs (Simard et al. 2013; Armes et al. 2009). Even though the problem is frequently encountered in clinical practice, no clear consensus exists on the best management strategies due to the scarcity of evidence-based therapies. This makes FCR a challenging problem for many survivors and health care providers (Thewes et al. 2014).

### Interventions for FCR

A more detailed description of intervention studies that have specifically addressed FCR (or related constructs) can be found in Additional file 1: Appendix A. Only one trial specified FCR as primary outcome of interest. Lebel et al., (2014) published a feasibility and preliminary outcome study of a single-arm 6-week cognitive existential group intervention to address moderate to high FCR in breast- and gynaecological cancer survivors. A decrease in FCR was found immediately following completion of the therapy and this effect was sustained at the 3-month follow-up. In 71% of the cancer survivors FCR could be classified as reliably improved and none of the cancer survivors showed deterioration (Lebel et al. 2014).

A construct that shares some defining features with FCR is fear of progression (FoP), the fear that the disease will further spread or progress in the body. A trial by Herschbach et al. (Herschbach et al. 2010) compared the effect of two four-session group interventions (cognitive behaviour therapy vs. supportive-experiential group therapy) and usual care on reducing dysfunctional FoP (Herschbach et al. 2010). Both interventions were carried out during cancer rehabilitation. FoP decreased significantly over time (up to 12-month follow-up) in both intervention groups in contrast to the control group. Those with metastatic disease or a recurrence (21%) benefitted most from the interventions (Lebel et al. 2014). A secondary analysis of this data by Sabariego et al., (2011) showed superior cost-effectiveness of group CBT over supportive-experiential group therapy for patients with high FoP (Sabariego et al. 2011).

Four intervention studies to improve generic emotional outcomes in breast cancer survivors addressed FCR as secondary measure (see Additional file 1: Appendix B). Two studies by Lengacher et al. 2009, 2011 investigated short-term effects of group mindfulness-based stress reduction (MBSR) on psychological status in breast cancer survivors. In the first study, a randomised controlled trial (RCT), breast cancer survivors participating in a 6-week MBSR programme reported a significant reduction in FCR over time compared to breast cancer survivors on a waitlist control group Lengacher et al. 2009 A second study by Lengacher et al., (2011) found significant improvement in FCR after completion of an 8-week MBSR programme (Lengacher et al. 2011). Both studies have only assessed short-term effects of the intervention and no information on long-term efficacy is available (Lengacher et al. 2009; Lengacher et al. 2011). The third study, a non-randomised trial, reported a significant decline in levels of FCR (cancer worry) following a 12-week emotion regulation group intervention targeted at anxiety and distress in breast cancer survivors. Yet, no long-term beneficial effects were found at 6-month or 12-month follow-up (Cameron et al. 2007). Finally, a brief self-guided nurse delivered uncertainty management intervention found no significant differences between the intervention and control group (Mishel et al. 2005). We are aware of two separate intervention trials for FCR currently in progress: Conquer Fear (Butow et al. 2013) and the AFTER-intervention (Humphris & Ozakinci 2008). Results of these studies have not yet been published.

To summarize, published intervention studies provide promising results in terms of beneficial effects of psychological interventions for FCR and related constructs. However, these studies had some limitations. While literature shows that moderate to high FCR is a universal problem in cancer survivors interventions have almost solely focused on survivors with breast- or gynaecological cancer (Herschbach et al. 2010; Lengacher et al. 2009; Lengacher et al. 2011; Mishel et al. 2005). Furthermore, information on treatment efficacy for long-term cancer survivors is limited because FCR was mainly addressed during the first year after diagnosis (Herschbach et al. 2010; Lengacher et al. 2009; Lengacher et al. 2011). Therefore, it is hard to generalise findings of efficacy and feasibility beyond women’s cancers to other cancer types, to men and to long-term cancer survivors. Only two studies mention screening for high FCR as part of their standard eligibility procedure (Lebel et al. 2014; Herschbach et al. 2010). By screening for high (dysfunctional) FCR, it is possible to identify those with the highest care need and to select those who might benefit most from the intervention. The SWORD-study was developed to address above mentioned limitations.

### Current study

This paper describes the development of the intervention and study protocol for the SWORD-study (SWORD is the acronym for Survivors’ Worries of Recurrent Disease). In this study an intervention known as “Beyond Fear” will be evaluated with regard to its efficacy in managing high FCR among breast, colorectal and prostate cancer survivors. In an RCT both the short-term and long-term effects of individual CBT for FCR will be investigated.

**Figure 1:**
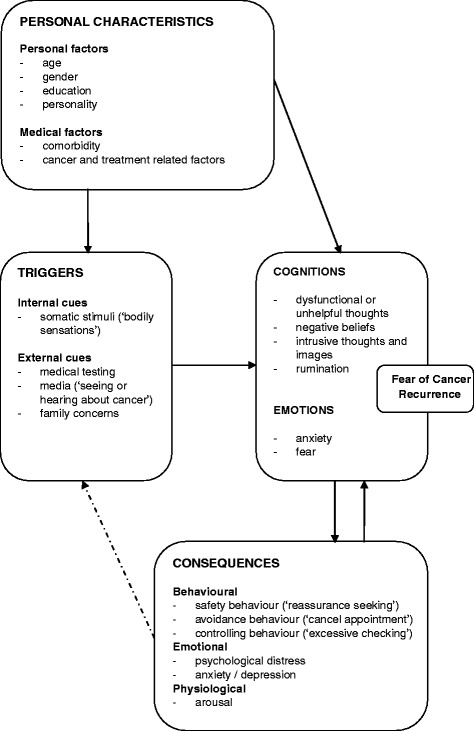
Theoretical model fear of cancer recurrence.

Our intervention expands on the theoretical formulation of FCR as a multidimensional construct proposed by Lee-Jones (1997; Lee-Jones et al. 1997). We updated this original formulation with recent research findings and clinical experience. According to our framework as shown in Figure 1, FCR is a distressing emotion maintained by dysfunctional cognitive thinking patterns such as recurring unhelpful thoughts, negative (illness) beliefs, intrusive images or persistent rumination. These thinking patterns cause a person to interpret certain events or internal stimuli as potentially threatening or harmful to one’s physical health and wellbeing, thereby triggering FCR. Consequently, behavioural strategies that are intended to reduce the fear, such as avoidance, excessive self-monitoring or safety-seeking behaviours, maintain FCR by preventing change in cognitive appraisal and/or by providing further exposure to triggers of FCR. While these behaviours may provide short-term alleviation of fear, they may actually maintain the fear on the long run (Lee-Jones et al. 1997; Leventhal et al. 1980). CBT targets high FCR by changing dysfunctional thinking patterns and maladaptive behaviours as specified in this model.

### Aims

The primary aim of the SWORD-study is to evaluate the efficacy of a combination of online and face-to face cognitive behaviour therapy (blended CBT) in reducing the impact of FCR in breast, colorectal and prostate cancer survivors. The aim is not to remove all FCR, but rather to reduce its severity in order to improve quality of life.

## Methods/design

The SWORD-study design and intervention are described conform the CONSORT guidelines for evaluation of randomised controlled trials (Schulz et al. 2010) and conform the CONSORT extension for non-pharmacological treatment interventions (Boutron et al. 2008).

### Trial design

The SWORD-study is a longitudinal, multicentre, two-arm, randomised controlled trial with one intervention condition (CBT) and one control condition; treatment as usual (TAU). Four assessments will take place for both trial conditions: baseline (T0, before randomisation), 3 (T1), 9 (T2) and 15 months (T3) after the baseline assessment. For survivors in the intervention condition, the CBT will take place between the first and second assessment. The longitudinal design allows for the assessment of both short-term and long-term effects of the intervention.

### Ethical consideration

The SWORD-study has been approved by the ethical review board of the Radboud University Medical Center (CMO Arnhem-Nijmegen). Approval of local ethics has been obtained in centres where recruitment will take place. Only survivors who have completed written informed consent will be allowed to participate. The study will be conducted in compliance with the guidelines for Good Clinical Practice and the Declaration of Helsinki (Pieterse 2010; World Medical Association & Declaration of Helsinki 2013). This trial is registered in the Netherlands National Trial Register (trial number NTR4423).

### Participants and procedure

A total of 104 (breast, prostate or colorectal) cancer survivors with high FCR are to be enrolled in this study. They will be randomly allocated to receive either CBT (n = 52, intervention condition) or TAU (n = 52, control condition). Cancer survivors will be recruited from outpatient clinics at an academic centre and several general hospitals in the Netherlands. Recruitment will take place at all sites simultaneously until the desired sample size is reached. Nurse practitioners are asked to provide an envelope containing study information (and an entry form) to all cancer survivors who are eligible for study participation based on information from their medical record. If interested to participate, a person will complete the entry form at home and send it to the researcher (MW) who will then contact them by phone to provide further study information and to address questions. Those willing to participate are asked to provide written informed consent and to fill-out a screening questionnaire. After receipt of the completed questionnaire, the researcher will contact the patient once more to discuss the screening outcome and to second check all eligibility criteria.

### Eligibility

Cancer survivors will be eligible to participate if they: (1) have completed primary treatment (with curative intent) for breast, colorectal or prostate cancer at least 6 months and not more than 5 years ago; (2) are disease-free at the moment of study inclusion, as defined by the absence of somatic disease activity parameters; (3) are at least 18 years of age; (4) score ≥ 14 on the Cancer Worry Scale, indicating high FCR; (5) have sufficient Dutch language skills to fill out questionnaires, to understand written text and to engage in active conversation; (6) are able to travel to the Radboud University Medical Center (RUMC) for CBT; (7) have given written informed consent. Cancer survivors are not eligible to participate if they 1) already receive psychological/psychiatric treatment at moment of inclusion; or 2) have a second primary tumour at moment of inclusion.

### Sample size

The sample size is calculated for the primary outcome FCR as measured with the Cancer Worry Scale (Custers et al. 2014). To detect a medium difference in FCR (Cohen’s d = 0.50), with a two-sided type I error rate of 0.05 and a power of .80, a sample size of 128 patients is needed. To correct for the baseline measurement as covariate the sample size is multiplied with the factor (1-r^2^), where r denotes the correlation between the baseline and post-intervention FCR (0.6 based on preliminary research) (Heinrichs et al. 2012). Therefore, a total sample size of 82 patients is desired. Because of an anticipated attrition rate of 20%, 52 patients are required per condition (104 total) (Lebel et al. 2014).

### Randomisation

After completion of baseline assessment survivors will be allocated to CBT or TAU according to a computer generated randomisation list, with a 1:1 allocation ratio using a fixed block size of six participants. Stratification by cancer type (breast, colorectal and prostate cancer) will be applied Randomisation is computerised, using a randomisation website specifically designed for this study. An independent secretary will enter all necessary patient data into the programme and will communicate the randomisation outcome (CBT or TAU) to the researcher who further informs the study participant. Cancer survivors allocated to the CBT condition will be assigned to one of two therapists according to therapist availability.

## Intervention

### Developmental process

The development of the intervention consisted of five stages:

● *Needs assessment:* Breast cancer survivors (n = 130) were approached with a ‘need for help’ question and the Cancer Worry Scale (CWS) (Custers et al. 2014). All survivors were asked if they would accept CBT specifically focused on managing FCR when experiencing high FCR (response options: yes/possibly/no). They were also provided with a short explanation of the CBT-content and therapy outline. Eighty-seven survivors completed both the ‘need for help’ question and the CWS. The majority (74%) of the responders considered or expressed a need for CBT (44% possibly; 30% yes). Almost the same response pattern was seen in those with high FCR (35% possibly; 30% yes) and those with low FCR (50% possibly; 30% yes) as based on the CWS. This procedure was replicated amongst 86 colorectal cancer survivors. Fifty-six percent of these survivors considered or expressed a need for CBT (38% possibly; 18% yes). Colorectal cancer survivors with high FCR were more open to CBT compared to survivors with low FCR (Low FCR, 38% possibly; 10% yes: High FCR, 39% possibly, 32% yes). Thus, a substantial number of cancer survivors indicate a need for help with FCR.

● *Content and structure of the intervention.* The intervention was developed by a collaboration of three clinical psychologists (JP/HK/PS), two researchers (MG/MW) and a psychiatrist (AS), all experienced in the field of psychosocial oncology and working at the RUMC. After literature consultation and multiple meetings, consensus was reached on core components and key techniques of the intervention (described in the section ‘Intervention: Cognitive Behaviour Therapy for FCR’).

CBT is one of the best established interventions for psychological problems in somatic conditions. In health care settings, CBT is already frequently used for various somatic problems such as fatigue and insomnia (Savard et al. 2005; Gielissen et al. 2006). CBT is a structured, action-oriented form of psychological treatment consisting of techniques directed at identifying and modifying negative (dysfunctional or unhelpful) thought patterns and dysfunctional behaviours (Beck & Beck 2011). Since cognitions, emotions and behaviours are interconnected, a change in cognitions and/or behaviour initiates changes in the other areas as well.

The intervention will be offered as blended therapy. In blended therapy both online and offline therapy components are integrated. A website is available that supports the patient throughout the entire therapy as it runs parallel to the face-to-face therapy sessions. The website has been developed in collaboration with ICT specialists and contains over 70 pages of content including information (10 scripts), at-home assignments (27 tasks), assessments (6 tests), audio (2 clips) and video (15 fragments). An incorporated library includes additional information on cancer-related topics. A feature to engage in an electronic consultation (‘e-consult’) with the assigned therapist is supplementary to the face-to-face sessions. Because not all survivors have access to the internet and some may lack the required computer skills, a paper workbook (with DVD/CD) will be available as well.

● *Advisory Committee.* The third step was to involve health care workers and patient representatives as an advisory committee in reviewing the therapy content. The committee was composed of three cancer survivors (breast, colorectal and prostate cancer) and three health care workers (two nurse practitioners for breast cancer and colorectal cancer care and a urologist). They were asked to provide comments, ideas and suggestions to further improve the intervention. In addition, a 13-item close-ended questionnaire, answerable on a 5-point Likert Scale (e.g. “*Overall, what is your general impression of the website?”*) was completed and members were asked to elaborate on their assigned score. The content and format of the intervention were rated with a mean of 4.3 out 5 (a higher score indicating a more positive impression of the intervention). With the generated feedback the content was slightly revised.

● *Website usability testing*. The user interface of the prototype website was tested by three patient representatives (breast, colorectal and prostate cancer) on feasibility and patient centeredness. A ‘think aloud procedure’ was employed, meaning that persons were asked to think aloud while using the website in order to provide us with more insight in 1) how the website is used without guidance of a professional and 2) how encountered problems are solved (Jaspers 2009). Afterwards the System Usability Scale (SUS) (Brooke 1996; Bangora et al. 2008) and a feedback form were completed. The SUS is a short (10-item scale) which gives a global view of subjective perception of usability, the mean total SUS score (range 0 - 100) given by the three survivors was 87, which indicates a satisfactory level of perceived usability (Bangora et al. 2008).

● *Pilot testing.* Lastly, two therapists piloted the intervention with two high fearful breast cancer survivors who completed all therapy sessions. After some minor revisions, the final content of the therapy manual was established.

### Intervention: cognitive behaviour therapy for FCR

The intervention was developed as face-to-face CBT with access to a website that provides online materials and the option to engage in therapist-patient interaction. For the therapists, a structured manual with a detailed description of each therapy session has been developed. The CBT covers 3 months and comprises five individual one-hour face-to-face sessions (session 1, 2, 3, 5 and 8) and three (15 minutes) e-consults or telephonic consultations (session 4, 6 and 7). In order to sustain behaviour change and monitor therapy progress, patients will be invited for a booster session at 3-month follow-up.

Intervention components were partially based on existing traditional CBT models for health anxiety and generalised anxiety. As with other forms of CBT, the primary emphasis of the therapy is on perpetuating factors of the problem in question. In this case, those factors maintaining high FCR. The principal therapeutic techniques are self-monitoring, cognitive restructuring (identification and re-attribution of unhelpful thoughts) and exposure- and response prevention. Other techniques are psychoeducation, relaxation, mindfulness, reframing, modelling (patient videos), at-home assignments, goal planning and attainment (Marks et al. 2011). The first session is directed at case conceptualisation and formulation of a personal FCR model, taking into account personal characteristics, triggers of fear, cognitions and consequences of FCR (see Figure 1). The FCR model guides the course of therapy by identifying the most appropriate points or targets for intervention (e.g. unhelpful cognitions). It is open to modification in the course of therapy because new insights might require adjustments in certain parts of the FCR model. The following four sessions (session 2 to 5) focus on acceptance, on identifying/modifying dysfunctional thinking patterns and on behaviour modification. If desired by patient or therapist, spouses will be invited to attend one or more therapy sessions. The final three sessions (session 6 to 8) are directed at consolidation of therapy progress and the establishment of a relapse prevention plan. Self-management skills are reinforced and active contribution to therapy progress and goal setting is encouraged. Completion of at-home assignments is of pivotal importance in order to practice the skills learned during therapy and to establish enduring change. A more detailed description of the intervention by session is described in Table 1.

**Table 1:** **Content of the intervention by therapy session**

**Session**	**Delivery**	**Week**	**Time (minutes)**	**Session components**
1	Face-to-face	1	90	• Case formulation: a patient’s story.
				• Discuss therapy rationale.
				• Establish therapy goals.
				• Review FCR and complete a personal FCR model.
				• Introduce at-home assignments.
2	Face-to-face	2	60	• Explain the basic tenets of CBT.
				• Discuss and visualize the association between thoughts, feelings and actions.
				• Review the concept of helpful beliefs.
				• Practice in filling out thought records.
				• Introduce mindfulness and relaxation exercises.
3	Face-to-face	3	60	• Review the completed thought record(s) to identify unhelpful thoughts and behavioural consequences of FCR.
				• Differentiate realistic from unrealistic worries and establish more helpful thoughts.
				• Explore and identify dysfunctional behavioural patterns.
				• Create a ranked list of situations that induce FCR and propose a behavioural experiment.
				• Practice a mindfulness or relaxation exercise.
4	E-consult (or telephone)	4	15	• Review of progress (troubleshooting).
				• Encourage at-home skill practice.
5	Face-to-face	6	60	• Review therapy goals, discuss areas of concern and make future plans (beyond therapy).
				• Discuss completed thought records and/or behavioural experiments.
				• Identify personal strengths and resources of strength.
6	E-consult (or telephone)	7	15	• Review of progress (troubleshooting).
				• Encourage at-home practice of skills.
7	E-consult (or telephone)	9	15	• Review of progress (troubleshooting).
				• Introduce the relapse prevention plan.
8	Face-to-face	11	60	• Review therapy goals, progress made so far and discuss possible future pitfalls.
				• Define and finalize the relapse prevention plan.
				• Evaluate the therapy process.
				• Schedule an appointment for the booster session.
9	Face-to-face (booster session)	24	60	• Review the FCR model and progress made during therapy.
				• Discuss difficult situations and how to overcome them.
				• Relapse prevention plan.

### Control condition: treatment as usual

Cancer survivors in the control condition have access to TAU and will not be offered additional psychological therapy for managing FCR. This condition reflects the natural course of FCR over time and gives insight in the standard care practices that are offered to persons outside the study context (e.g. during routine medical follow-up). In the period after cancer treatment all patients are offered medical follow-up appointments conform the recommendations of the Dutch guidelines in oncology care (Comprehensive Cancer Centre the Netherlands 2011). For colorectal cancer, the Dutch guideline advises medical examinations every 6 months during the first 2 years of follow-up, continued by yearly examinations up to 5-years follow up. For breast cancer, the Dutch guideline advises medical follow-up examinations every 3 months during the first year, every 6 months during the second year and examinations once a year during 2 to 5 years follow-up. For prostate cancer, during the first year after cancer treatment a follow-up schedule of 6 weeks, 3, 6, 9 and 12 months is recommended, and semi-annually or annually thereafter for 5 to 10 years. In the Netherlands, psychosocial follow-up is not institutionalised and psychosocial care offered to cancer survivors with high FCR therefore differs between health care institutions. Information on additional medical or psychosocial care survivors have had during the study period will be collected for both the interventions and the control group. This includes utilization of psychosocial services (e.g. psychological therapy, mindfulness and social work), health care consultations (e.g. GP, medical specialists, and paramedical assistance) or medication use.

### Participating therapists

All CBT sessions will take place at the RUMC, department of Medical Psychology in Nijmegen, the Netherlands. The CBT will be practiced by two qualified, registered healthcare psychologists with experience in delivering CBT for somatic conditions and experience in the field of psychosocial oncology. During the study, both therapists will have regular supervision by a registered clinical psychologist, also qualified as CBT supervisor (JP). Before the start of the study, both therapists had already performed one supervised treatment case conform the therapy manual.

### Treatment integrity

To be able to draw valid conclusions on the therapy effects, treatment integrity (e.g. the implementation of the treatment as intended) is ensured conform the guidelines established by the Behaviour Change Consortium (Bellg et al. 2004), i.e. with the use of a standardised therapy manual and ongoing therapist supervision. All sessions will be audio taped and 5% will be randomly checked for adequate therapy implementation.

### Outcomes

Detailed information on the study outcomes is available in Table 2. Participants will be asked to complete questionnaires at four different time points, either online or on paper. Demographic and medical information will be gathered with self-report questionnaires and from medical records.

**Table 2:** **Primary and secondary outcome measures of the SWORD-study**

**Primary outcome**				
	**Questionnaire**	**Response format**	**Example question**	**Timepoints**
**Fear of cancer recurrence**	Cancer Worry Scale (CWS) (8 items)	4-point Likert scale *range 8 - 32	*During the past month: “How often have you thought about your chances of getting cancer (again)?”*	Screening T0;T1;T2;T3
**Secondary outcomes**				
**Dimensions of FCR**	Fear of Cancer Recurrence Inventory (42-items)	5-point Likert scale	*“How many times per day do you spend thinking about the possible chance of recurrence?” “I am worried or anxious about a possible chance of recurrence.” “I try to replace this thought with a more pleasant one.”*	T0;T1;T2;T3
	Subscales:			
	• Triggers (8 items)	−0 - 32		
	• Severity (9 items)	−0 - 36		
	• Psychological distress (4 items)	−0 - 16		
	• Functional impairment (6 items)	−0 - 24		
	• Coping strategies (9-items)	−0 - 36		
	• Insight (3 items)	−0 - 12		
	• Reassurance Seeking (3 items)	−0 - 12		
**Cancer related quality of life**	EORTC-QLC C30 (30 items) Subscales:	4-point Likert scale	*“Were you limited in pursuing your hobbies or other leisure time activities?” “How would you rate your overall health during the past week? (Global health)”*	T0;T1;T2;T3
	• Five functional scales (15 items)	−15 - 60		
	• Three symptom scales (7 items)	−7 - 28		
	• Single symptom items (6 items)	−6 - 24		
	• Global health & quality of life scales (2 items)	−2 - 14		
**Cancer specific quality of life: Breast cancer**	EORTC-BR23 (23 items) Four functional scales (8 items)	−8 - 32	*“Have you felt physically less attractive as a result of your disease or treatment?”*	T0;T1;T2;T3
	• Four symptom scales (15 items)	−15 - 60		
**Cancer specific quality of life: Prostate cancer**	EORTC-PR25 (25 items)		*“Have you had difficulty going out of the house because you needed to be close to a toilet?”*	T0;T1;T2;T3
	• Two functional scales (6 items)	−6 - 24		
	• Four symptom scales (19 items)	−19 - 76		
**Cancer specific quality of life: Colorectal cancer**	EORTC-CR38 (38 items) Two functional scales (7 items)	−7 - 28	*“During past week, where you bothered by gas (flatulence)?”*	T0;T1;T2;T3
	• Seven symptom scales (28 items)	−26 - 104		
	• Three single symptom items	−1 - 4		
**Satisfaction with life**	Satisfaction With Life Scale (5-items)	7-point Likert scale *5 - 35	*“In general, I am satisfied with life.”*	T0;T1;T2;T3
**Distress**	Hospital Anxiety and Depression Scale (14 items) Subscales	4-point Likert scale *0 - 42	*“I feel tense or wound up” “I feel as if I am slowed down”*	T0;T1;T2;T3
	• Anxiety (7 items)	−0 - 21		
	• Depression (7 items)	−0 - 21		
	Distress thermometer (1 item) Problem List (47 items)	VAS *0 - 10 *0 - 47	*“Circle the number (0-10) that best describes how much distress you have been experiencing in the past week.”*	T0;T1;T2;T3
**Fatigue severity**	Checklist Individual Strength – Fatigue Severity subscale (8 items)	7-point Likert scale *8 - 56	*“I feel tired” “I am rested”*	T0;T1;T2;T3
**Optimism**	Life orientation Test (LOT) (12 items)	5-point likert scale *0 - 32	*“In uncertain times, I usually expect the best.”*	T0;T1;T2;T3
**Body vigilance**	Body Vigilance Scale (4-items)	VAS scale *0 - 10 item 1-3 *0 - 15 item 4	*“I am very sensitive to changes in my internal bodily sensations”*	T0;T1;T2;T3
**Coping with the experience of cancer**	Impact of Events Scale (15 items) Subscales	4-point Likert scale *0 - 35 *0 - 40	*“Any reminder brought back feelings about it.” (intrusion)*	T0;T1;T2;T3
	• Intrusion (7 items)			
	• Avoidance (8 items)			
**Perceived social support**	Social Support List – Dissatisfaction scale (34-item)	4-point likert scale *34 - 102	*What is your opinion about the extent to which people…: “Drop in for a pleasant visit?”*	T0;T1;T2;T3
**Personality**	Big Five Inventory (44 items) Subscales Neuroticism (8 items)	5-point Likert scale	I see myself as someone: *“Who… can be somewhat careless.” (conscientiousness) “Who… Is sometimes shy, inhibited.” (extraversion*)	T0
	• Extraversion (8 items)			
	• Openness (10 items)			
	• Conscientiousness (9 items)			
	• Agreeableness (9 items)			
**Health care use**	EQ-5D (5 items) EQ-5D thermometer	4-point Likert scale VAS Scale (0 - 100)	*“Please indicate which statement best describes your own health state today.”*	T0;T1;T2;T3
	TIC-P – part I	Open ended questions	*In the past 4 weeks did you “how often did you visit the general practitioner?”*	T0
	Custom made cost diaries	Open ended questions	*During the specified period: ‘How often did you consult the general practitioner and why?’*	Between T0-T1 T1-T2 T2-T3 T3-T4

### Screening and primary outcome

Participants will be screened on high *Fear of cancer recurrence* with the Cancer Worry Scale (CWS). This questionnaire is validated as a screening instrument and is able to detect high FCR in Dutch cancer survivors (Custers et al. 2014). A cut-off score of ≥ 14 appeared optimal for differentiating high fearful patients from non-fearful patients. The CWS has good psychometric properties (α = 0.87).

### Secondary outcomes

*Multidimensional aspects of FCR* will be assessed with the Fear of Cancer Recurrence Inventory (FCRI). The FCRI gives a global idea of FCR in the preceding month and provides information about the principal characteristics of FCR (e.g. fear invoking stimuli/situations). The FCRI consists of 7 subscales; Triggers, Severity, Psychological distress, Coping strategies, Functioning Impairments, Insight, and Reassurance (Simard & Savard 2009).

*Quality of Life* will be measured with the Dutch version of the European Organization for Research and Treatment of Cancer (EORTC) Quality of Life Questionnaire Core 30 (QLQ-C30). Complementary to the QLQ-C30, disease specific modules for breast cancer (QLQ-BR23), colorectal cancer (QLQ-CR38) and prostate cancer (QLQ-PR25) will be assessed (Aaronson et al. 1993). Both the EORTC-QLQ-C30 and the disease specific modules have demonstrated moderate to good psychometric properties and clinical validity in cancer survivors (Aaronson et al. 1993; Sprangers et al. 1996; Sprangers et al. 1999; van Andel et al. 2008).

*Satisfaction with life* will be evaluated with the Satisfaction With Life Scale (SWLS). The SWLS has sufficient psychometric properties (α = .87) and is able to detect changes in life satisfaction over time (Diener et al. 1985).

*Distress* will be measured with the Hospital Anxiety and Depression Scale (HADS) total score (Annunziata et al. 2011; Vodermaier & Millman 2011). In addition, the Dutch version of the Distress Thermometer (DT) and the problem list will be completed as well (Tuinman et al. 2008).

*Fatigue* severity will be assessed with the fatigue severity subscale of the Checklist Individual Strength (CIS-8R) (Dittner et al. 2004; Servaes et al. 2002).

*Bodily vigilance* refers to the tendency to focus on internal bodily sensations and will be assessed with the four-item Body Vigilance Scale (Schmidt et al. 1997).

*Coping with the experience of cancer* will be measured with the Impact of Events Scale. This scale consists of 15 items that ask for the frequency of cancer-related avoidant and intrusive cognitions or behaviours (Horowitz et al. 1979) .

*Perceived social support,* or rather the perceived discrepancy between a patient’s desired social support and the actual amount of social support received, will be assessed with the Social Support List – Dissatisfaction (SSL-D) scale (Van Sonderen & Ormel 1997).

*Personality* dimensions will be measured with the Big Five Inventory (BFI) (Denissen et al. 2008). This is a 44-item multidimensional personality inventory that covers the five main dimensions of personality trait (conscientiousness, agreeableness, emotional stability, extroversion and intellect or openness (John & Srivastava 1999)).The BFI will only be administered at baseline (T0).

*Optimism*, as a dispositional trait, will be assessed with the Life Orientation Test (LOT). This questionnaire contains twelve items on the optimistic and pessimistic trait of personality (Scheier & Carver 1985).

*Health Care Use/cost-effectiveness:* The EuroQol-5D (EQ-5D) will be used to calculate *cost-utility.* It has shown to be an appropriate measure for economic evaluations in health interventions with breast cancer patients after treatment (Kimman et al. 2009). The instrument is able to detect changes in patients’ self-reported health related quality of life and has good psychometric properties (Kimman et al. 2009; Ravens-Sieberer 2010). *Health care costs* will be further monitored with cost diaries and the Trimbos/iMTA Costs associated with Psychiatric Illness (TiC-P) questionnaire (Hakkaart-Van Roijen 2002). Patients will be asked to report both direct health care costs (e.g. use of health care services, change of prescribed medication) and indirect costs (e.g. absence from work) during specified time periods (see Table 2).

### Technical usage statistics (intervention condition only)

Website use and completion of exercises can be seen as a form of treatment adherence. This includes data on number of exercises completed, frequency of logins, time online and webpages opened (Donkin et al. 2011). Technical data for those using the workbook will include the number of exercises completed.

### Intended statistical analyses

SPSS will be used for all statistical analyses. All statistical tests will be two-sided (level of significance = 0.05). To ensure that the key variables are evenly distributed by randomisation, baseline characteristics will be compared between the two conditions with Chi-square (categorical variables) and ANOVA (continuous variables) testing. Primary efficacy analysis will be conducted in agreement with the intention-to-treat principle. Additionally, a per-protocol analysis will be conducted using the data for those who successfully completed the intervention. Differences between the two conditions in the amount of change in FCR (T0 and T1) will be calculated with ANCOVA-analysis. Later, the follow-up effects (T3, T4) will be investigated with longitudinal data analysis. Next to statistical significance the clinically significant improvement will be established according to the method by Jacobson & Truax, calculating the reliable change index (Jacobsen & Truax 1984).

## Discussion

While cancer survivors report FCR to be a key concern and unmet need no clear consensus exists on the best management strategies for FCR in clinical practice. The SWORD-study protocol describes a trial that will evaluate blended CBT as intervention for high FCR in cancer survivors. The primary aim is to reduce high FCR to more acceptable and less debilitating levels. Both post-intervention and follow-up effects of the therapy will be assessed. If efficacy can be demonstrated, an evidence-based therapy for high FCR will become available.

Strengths of this study include: an intervention specifically targeted at high FCR, the inclusion of breast, colorectal and prostate cancer survivors, screening for high FCR and the inclusion of survivors up to five years post medical treatment. Until now, only one published (feasibility) trial investigated an intervention specifically targeted at high FCR. Other studies have mentioned FCR as a secondary outcome or have investigated related constructs (e.g. FoP). Furthermore, because interventions were mostly targeted at survivors of breast or gynaecological cancer there was an overrepresentation of women in all samples (88-100% women). It is assumed that men and women experience and deal with emotional problems differently. Women are more comfortable in disclosing their feelings than men and might be more inclined to seek and accept expert help (Northouse et al. 2000). It is therefore debatable whether comparable intervention effects can be achieved in male cancer survivors. In order to overcome this problem, our trial includes survivors of male (prostate), female (breast) and mixed gender (colorectal) cancer.

FCR is a concern for many cancer survivors, ranging from mild to high levels. Hence, different forms of care, each with different intensity, should be available to cancer survivors based on their individual needs (Bower & Gilbody 2005). The first level of tailored care for milder FCR may only comprise of patient education or psycho-education while survivors with moderate FCR could benefit from a more intensive approach such as empowering self-management through internet therapy. High specialised care can comprise individual CBT, of which our intervention would be a good example. In order to identify those who might benefit most from blended CBT screening for high FCR will take place and those scoring above the specified threshold (CWS ≥14) will be asked to participate.

To conclude, the current trial will answer the question whether blended CBT is an effective intervention to manage high FCR in breast, prostate and colorectal cancer survivors. If so, an evidence-based therapy to manage high FCR will become available for use in clinical practice.

## Additional file

Additional file 1:
**Appendix A.** Interventions specifically targeted at FCR (or FoP). **Appendix B.** Interventions that mention FCR as secondary outcome.

## Abbreviations

FCR: Fear of cancer recurrence
CBT: Cognitive behaviour therapy
FoP: Fear of progression
RCT: Randomised controlled trial
MBSR: Mindfulness-based stress reduction
TAU: Treatment as usual
RUMC: Radboud University Medical Center
