# Supplementary material for: Study protocol of the SWORD-study: a randomised controlled trial comparing combined online and face-to-face cognitive behaviour therapy versus treatment as usual in managing fear of cancer recurrence
Source: BMC Psychol. 2015 Apr 16;3(1):12. doi: 10.1186/s40359-015-0068-1 (PMC4431367; doi:10.1186/s40359-015-0068-1)
Supplement: Additional file 1: — Appendix A. Interventions specifically targeted at FCR (or FoP). Appendix B. Interventions that mention FCR as secondary outcome. [file 40359_2015_68_MOESM1_ESM.pdf]

*Appendix A. Interventions specifically targeted at FCR (or FoP)*

| **Study** | **Primary outcome** | **Screening FCR** | **Design** | **Sample** | **Intervention conditions and descriptions** | **Findings** |
| --- | --- | --- | --- | --- | --- | --- |
| Lebel, 2014 | FCR | Yes. FCR score > 4 on 50% of the 22 items of the fear of recurrence questionnaire + IES score ≥26. | Longitudinal, single-arm design (feasibility study).  Assessments: baseline (T0), post-treatment (T1) and 3-month follow-up (T2). | *n* = 56 cancer survivors.  Breast (*n* = 46) and gynaecological (*n* = 10) cancer. Stage I-III disease. Only women.  Time since diagnosis: All survivors had previously completed primary treatment. (Diagnosed M2.3 years earlier).  Age: 54.8 years (SD 9.0). | Cognitive-existential group intervention (*n* = 56).  Six consecutive weekly group sessions of 90 minutes each. Total of nine groups (two for ovarian cancer, seven for breast cancer).  Components of existential psychotherapy focusing on subjective experiences, facing existential anxiety and explore meaningfulness of life. | A significant reduction in FCR was found (T0-T1). This change was sustained at follow-up (T2).  Secondary outcomes:  ↓ Cancer specific distress  ↓ Uncertainty  21% attrition |
| Herschbach, 2010  Sabariego, 2011 | FoP  Cost-effectiveness | Yes. FoP is > cut-off score on the FoP-Q-short form. | Longitudinal controlled study.  Two intervention groups vs. one control group.  Assessments: baseline (T0), post-treatment (T1), 3- and 12-month follow-up (T2 and T3).  *Control group was recruited one year after study start. This group completed T0, T1 and T3.*  Cost-effectiveness analysis of study by Herschbach et al., (2010). | *n* = 265 mixed cancer sites. Breast (*n* = 155), colorectal (*n* = 21), bladder/prostate (*n* = 25), gynaecological cancer (*n* = 24) and other (*n* = 38).  All disease stages.  21% metastasized disease,  83% women.  Time since diagnosis: All survivors were in cancer rehabilitation.  Age: 53.7 years (SD 10.2).  See above. | 1. Cognitive Behavioural Group Therapy  (*n* = 91).  Four sessions of 90 minutes each and one booster session of 15 minutes (by phone) at 6 and 9 months after discharge from clinic. During therapy survivors learn to identify and recognize individual fears. Learn coping strategies. Total of 15 groups with four to ten participants each.  2. Supportive-Experiential Group Therapy (*n* = 85).  Four sessions of 90 minutes each and one booster session of 15 minutes (by phone) at 6 and 9 months after discharge from clinic. SET is a non-directed, client-centred intervention that has theme-centred interactions. Emotional experiences and social support are important aspects.  Total of 15 groups with four to ten participants each.  3. Control group (*n* = 91)  Cancer survivors offered no extra intervention during cancer rehabilitation. | All three conditions showed a short-term improvement in FoP (T0 to T1).  A long-term reduction (T3) in FoP was only found for the intervention conditions, not the control condition.  Secondary outcomes:  ↓ Depression  ↓ Anxiety  ↑ HrQoL  Finding:  Superior cost-effectiveness of CBT over SET for patients with  high levels of anxiety. |

| **Study** | **Primary outcome** | **Screening FCR** | **Design** | **Sample** | **Intervention conditions and descriptions** | **Findings** |
| --- | --- | --- | --- | --- | --- | --- |
| Humphreys, 2008 | FCR | Unknown | The intervention will be tested in a randomised controlled trial. | Head and neck cancer survivors. | The AFTER-intervention  Six individual therapy sessions of 60 minutes each. Intervention components are: emotional expression, behavioural management, cognitive restructuring, relaxation and caregiver incorporation. | Findings not yet available. |
| Butow, 2013 | FCR | Yes. Score of ≥13 on the FCRI severity scale. | The intervention is currently being tested in a randomised controlled trial.  One intervention group vs. one control group.  Assessments: baseline (T0), post-treatment (T1), 3- and 6-month follow-up (T2 and T3). | Breast cancer survivors (stage 0-2) and colorectal cancer survivors (Duke stage A-C). | 1. the Conquer-Fear intervention  Five individual face-to-face sessions of 60 – 90 minutes each over a period of 10 weeks. Sessions are delivered by a trained therapist. The intervention is based on meta-cognitive therapy and has components of acceptance and commitment therapy. The patient is taught more effective ways to respond to FCR and existential issues are addressed.  2. Relaxation training (control condition).  The therapy consists of five relaxation training sessions over a period of 10 weeks. | Findings not yet available. |

*Abbreviations: FCR: Fear of cancer recurrence, FoP: Fear of Progression, IES: Impact of Events Scale.*

*Appendix B. Interventions that mention FCR as secondary outcome.*

| **Study** | **Primary outcome** | **Screening FCR** | **Design** | **Sample** | **Intervention conditions and descriptions** | **Findings** |
| --- | --- | --- | --- | --- | --- | --- |
| Lengacher, 2009 | Psychological status | No | Randomised controlled trial, longitudinal study.  One intervention group vs. one control group.  Assessments*:* baseline (T0) and post-treatment (T1). | *n =* 84 breast cancer survivors. Stage 0 – III disease. Only women.  Time since diagnosis: all survivors were within 18 months of medical treatment completion.  Mean age: 57.5 years (SD 9.4). | 1. Mindfulness-Based Stress Reduction (*n* = 41)  Six weekly two-hour group sessions of MBSR, adjusted for breast cancer survivors. Survivors learn to self-regulate arousal to stressful circumstances or symptoms. MBSR includes mindful attention, yoga practices and meditational exercises. Total of seven groups with four to eight participants each.  2. Treatment as usual (*n* = 43)  A waitlisted control group offered no intervention during study period. | Improvement in terms of psychological wellbeing (T0-T1):  ↓ Depression  ↓ Anxiety  ↓FCR  ↑ Quality of life |
| Lengacher, 2011 | Psychological status | No | Single-group, pretest-posttest design (feasibility study)  Assessments: baseline (T0) and post-treatment (T1). | *n* = 19 breast cancer survivors. Stage 0 – I disease. Only women.  Time since diagnosis: all survivors were up to 1 year after medical treatment.  Mean age: 56.8 years (SD 8.8). | Mindfulness-Based Stress Reduction (*n* = 19).  Eight weekly group sessions of two-hours, adjusted for breast cancer survivors. Important treatment components: education, practice and group processes. Training in mediation techniques with emphasis on attention to breathing. | Improvements in terms of psychological wellbeing (T0-T1):  ↓ FCR  ↓ Anxiety  ↓Depression  ↓Perceived stress  ↑ QoL (emotional wellbeing and general health)  67% of all participants attended at least 6 classes (out of 8). |
| Mishel, 2005 | Uncertainty | No | Randomised Controlled Trial (pilot study).  One intervention group vs. one control group.  Assessments*:* baseline (T0) and 10-month post-treatment (T1). | *n* = 509 breast cancer survivors. Stage 0-III disease. Only women.  Time since diagnosis: all survivors were between 5 – 9 years after medical treatment.    Mean age: 64 years (SD 8.9). | 1. Uncertainty management intervention (*n* = 244)  Brief guided telephonic intervention with audiotapes, a self-help manual and 4 weekly telephone calls by a nurse. Main components: cognitive therapy to stimulate emotion-focused coping skills and behavioural strategies to learn self-management skills.  2. Control group (*n* = 265) | No uncertainty data available at T1.  ↑ Cognitive reframing  ↑ Cancer knowledge  ↑ Communication  ↑ Coping skills |
| Cameron, 2007 | Adjustment to breast cancer. | No | Longitudinal, quasi-experimental design with an alternating phases intervention.  One intervention group vs. two control groups.  Assessments: baseline (T0), post-treatment (T1), 6- and 12-month follow-up (T2 and T3). | *n* = 154 Breast cancer survivors. Only women.  Time since diagnosis: Diagnosed with breast cancer in the previous six weeks.  Mean age: ranging from 48.2 years (SD 8.53) in the intervention condition to 54.6 years (SD 8.94) in the decliner group. | 1. Emotion regulation intervention (*n* = 54).  The 12-week group intervention consisted of weekly two-hour sessions. Intervention components are relaxation, guided imagery, meditation and emotional expressing. Aim of the treatment is to alter emotion regulation and to promote adjustment to cancer.  2. Decliner group (*n* = 56)  Declined intervention participation.  3. Standard care (*n* = 44) | Improvements in terms of psychological wellbeing (T0-T1):  ↓ Cancer Worry  ↓ Anxious  ↑ Coping efficacy  ↑ Emotional wellbeing  Effects not sustained at follow-up (T2 or T3). |
